# Chirality of antidepressive drugs: an overview of stereoselectivity

**DOI:** 10.2478/abm-2022-0008

**Published:** 2022-04-29

**Authors:** Vinod Kumar Vashistha, Sonika Sethi, Inderjeet Tyagi, Dipak Kumar Das

**Affiliations:** Department of Chemistry, GLA University, Mathura, Uttar Pradesh 281406, India; Department of Chemistry, GD Goenka University, Gurgaon, Haryana 122103, India; Centre for DNA Taxonomy, Molecular Systematics Division, Zoological Survey of India, Ministry of Environment, Forest and Climate Change, Government of India, Kolkata 700053, India

**Keywords:** antidepressive agents, chiral, depression, pharmacokinetics, stereoselective

## Abstract

Stereochemistry plays an important role in drug design because the enantiomers of a drug frequently vary in their biological action and pharmacokinetic profiles. Racemates of a drug with either an inactive or an unsafe enantiomer can lead to detrimental effects. The manufacturing industry may still produce racemates, but such decisions must pass through rigorous analyses of the pharmacological and pharmacokinetic characteristics of the particular enantiomer related to the racemates. The pharmacokinetics of antidepressants or antidepressive agents is stereoselective and predominantly favors one enantiomer. The use of pure enantiomers offers (i) better specificity than the racemates in terms of certain pharmacological actions, (ii) enhanced clinical indications, and (iii) optimized pharmacokinetics. Therefore, controlling the stereoselectivity in the pharmacokinetics of antidepressive drugs is of critical importance in dealing with depression and psychiatric conditions. The objective of this review is to highlight the importance of the stereochemistry of antidepressants in the context of the design and development of new chirally pure pharmaceuticals, the potential complications caused by using racemates, and the benefits of using pure enantiomers.

Depression is one of the most common psychological illnesses, affecting >340 million people globally. This condition results in a 10% decrease of productive years, which cost the USA alone an additional US$210.5 billion in 2010, an increase of around 22% since 2005. Depressive disorders cause disability and are the single largest contributing group to nonfatal health loss worldwide [1, 2]. At any stage in life, people may experience sporadic feelings of anxiety or depression. Feeling depressed is a natural human response to challenging situations, such as the death of a loved one, financial difficulties, or personal issues. When such emotions transform into overwhelming sadness, or when a person feels hopeless, useless, or desperate, and if this lasts for a week or longer, the emotions might lead to a mood disorder and clinical depression. The symptoms of clinical depression impede everyday life and cause serious discomfort to the patients, their family, and friends. Although people with serious depressive symptoms can recover with appropriate treatment, many do not ever seek treatment because of the related societal stigma.

For reasons that are unclear, women and girls are nearly twice as likely to suffer from depression as men and boys. At any point in their life, close to 26% of women and girls suffer depression, by contrast with the figure among men and boys (up to 12%). Stress is among the greatest causes of depression, as shifts in certain aspects of life, society, professions, and ways of transport, in addition to drastic lifestyle changes, bring pressure on people to adapt at equal speed. In a representative population-based analysis using a medical expenditure panel survey, the proportion of those undergoing antidepressive treatment and those taking antidepressive agents through 2 decades (between 1996 and 2015) was calculated, and the prevalence of adults diagnosed with a major depressive disorder was found to have increased from 6.1% in 1996 to 10.4% in 2015 [2].

Depression is usually triggered by anomalies in the activities or production of certain neurotransmitters, particularly norepinephrine and dopamine. Norepinephrine controls alertness, attention, and motivation. Pharmacological treatments of depression vary widely among individuals.

Most antipsychotic and antidepressive medications are sold as a racemic mixture of their enantiomers, and some are available as individual enantiomers (**Table 1**). The response to these medications varies among individuals. Switching antidepressants is recommended when some patients have intolerable side effects, when the efficacy of the medication is low, or when there are adverse interactions between an antidepressant and other prescribed medications [3]. The enantiomers or geometric isomers of a drug molecule differ substantially from one another in terms of their pharmacological properties. The latest advances in enantiomeric resolution have increased understanding of the chemical structure of drug targets. Increased perception of the many possible benefits of using single enantiomers instead of racemic drug mixtures has resulted in an increased focus to comprehend the role that chirality plays in drug design [4,5,6]. This has led to improved analysis of single enantiomers during drug development and chiral switching, that is, replacing a racemate with a substance that is already registered or sold as a pure enantiomer. While stereochemistry is an essential aspect to consider in all drug development, this review emphasizes the importance of the chirality of antidepressive agents.

**Table 1:** Classification of chiral antidepressant drugs

**Class**	**Generic name**	**Brand name**	**Available as**	**More active form**
SSRI	citalopram	Celexa, Cipramil, Zetalo, Lexapro	Racemate and (*S*)-citalopram	(*S*)-citalopram
	fluoxetine	Prozac, Sarafem, Rapiflux	Racemate and (*S*)-fluoxetine	(*S*)-fluoxetine
	sertraline	Zoloft, Actiser, Bioserene, Episod, Mentolift	Racemate and (+)-*cis*-(1*S*,4*S*)-sertraline	(+)-*cis*-(1*S*,4*S*)-sertraline
SNRI	paroxetine	Paxil, Brisdelle, Paxil CR, Pexeva	(−)-*trans*-(3*S*,4*R*)-paroxetine	(−)-*trans*-(3*S*,4*R*)-paroxetine
	venlafaxine	Effexor, Effexor XR	Racemate	(*R*)-venlafaxine
	duloxetine	Cymbalta, Drizalma Sprinkle, Irenka	(*S*)-duloxetine	(*S*)-duloxetine
	milnacipran	Ixel, Savella, Dalcipran, Toledomin	Mixture of d-milnacipran (1*S*,2*R*) and l-milnacipran (1*R*,2*S*)	d-milnacipran (1*S*,2*R*)
NRI	reboxetine	Edronax, Pfizer	Mixture of (*S*,*R*)-reboxetine, (*R*,*R*)-, (*R*,*S*)-reboxetine (*S*,*S*)-reboxetine	(*S*,*S*)-reboxetine
NDRI	bupropion	Wellbutrin, Zyban	Racemate	(*R*)-bupropion
MAOI	selegiline	Eldepryl, Zelapar	Racemate	(+)-selegiline
TeCA	mianserin	Bonserin, Tolvon, Athimil, Depnon, Deprexolet, Lantanon, Lerivon, Demisone, and others	Racemate	(*S*)-mianserin
NASSA	mirtazapine	Remeron, Mirataz, and others	Racemate	(*S*)-mirtazapine

MAOI, monoamine oxidase inhibitor; NASSA, noradrenergic and specific serotonergic antidepressant; NDRI, norepinephrine–dopamine reuptake inhibitor; NRI, norepinephrine (noradrenaline) reuptake inhibitor; SNRI, serotonin–norepinephrine reuptake inhibitor; SSRI, selective serotonin reuptake inhibitor; TeCA, tetracyclic antidepressant.

Science Direct, PubMed, Google Scholar, and Scopus databases were used to gather data for this review. The major descriptors used were selective serotonin reuptake inhibitor (SSRI), antidepressants, stereoselectivity chirality of antidepressants, and pharmacokinetics.

During the past 2 decades, several important reviews of antidepressants have appeared; a summary of these reviews is provided below:

- Sethi and Bhushan [1] presented a review article on enantioselective liquid chromatography (LC) analysis and determination of SSRIs. The review highlights a range of separation techniques and related aspects of antidepressant drugs.
- Wei et al. [7] presented a review entitled “A historical review of antidepressant effects of ketamine and its enantiomers” in *Pharmacology, Biochemistry, and Behavior*, in which the authors discussed a history of (*R,S*)-ketamine and the effects of ketamine enantiomers in humans and rodents. The mechanisms of ketamine's antidepressive effects are discussed.
- “A brief history of antidepressant drug development: from tricyclics to beyond ketamine,” published in 2018, provides a retrospective account of monoaminergic antidepressant drug development [8].
- “Chirality of modern antidepressants: an overview,” by Budău et al. [9] is a review on the importance of chirality of a few modern antidepressive agents. However, there is no apparent discussion of their classification.
- “Stereospecific LC and LC-mass spectrometry (MS) bioassays of antidepressants and psychotics,” by Nageswara Rao and Guru Prasad [10], in 2015, reviewed LC methods for the analysis of chiral antidepressive agents. The review provides a discussion of the advancement of chromatographic methods, including both direct and indirect approaches reported from 2000 to 2013.
- “Stereochemistry and drug efficacy and development: relevance of chirality to antidepressant and antipsychotic drugs,” by Baker and Prior [11] in 2002 reviewed the role of each enantiomer in the efficacy of a drug. However, the discussion is limited to only a few antidepressive agents.
- “Enantiomeric antidepressant drugs should be considered on individual merit,” by Baumann and Eap [12] in 2001, reviewed the enantiomeric pharmacology of 5 chiral anti-depressant drugs. They highlighted the stereoselective characteristics of enantiomeric drugs.
- “Great expectations in stereochemistry: focus on antidepressants” by DeVane and Boulton [13], in 2002, highlighted the role of chirality in the stereoselective disposition and actions of antidepressants, although the focus was on a limited number of antidepressive agents.

Our present review comprises a discussion of various antidepressive agents listed in the sections as follows:

1. Selective serotonin reuptake inhibitors (SSRIs)
  (a) Citalopram
  (b) Fluoxetine
  (c) Sertraline
  (d) Paroxetine
2. Serotonin–norepinephrine reuptake inhibitors (SNRIs)
  (a) Venlafaxine
  (b) Duloxetine
  (c) Milnacipran
3. Norepinephrine (noradrenaline) reuptake inhibitors (NRIs)
  (a) Reboxetine
4. Norepinephrine–dopamine reuptake inhibitors (NDRIs)
  (a) Bupropion
5. Monoamine oxidase inhibitors (MAOIs)
6. Tetracyclic antidepressants (TeCAs)
7. Noradrenergic and specific serotonergic antidepressants (NASSAs)

## Classification of antidepressive agents

There are various categories of antidepressant drugs. Each category has a different action mechanism and profile of side effects. These have been described by Wasan et al. [14].

## Selective serotonin reuptake inhibitors

SSRIs are the most widely approved second-generation anti-depressant drugs used to relieve the symptoms of mild-to-serious depression. Compared with other forms of antidepressant drugs, these are reasonably safe with rare adverse effects. SSRIs counter depression by affecting neurotransmitters. Certain other disorders, including anxiety, obsessive–compulsive disorder, panic disorder, and phobic neurosis, are also controlled by SSRIs. Drug–drug interactions and therapeutic drug monitoring of non-SSRI antidepressants have been reviewed by Protti et al. [15]. These authors have discussed the metabolism and interactions of these drugs, and brief notes on analytical methods useful for therapeutic monitoring have also been discussed.

### Citalopram

**Figure 1:**
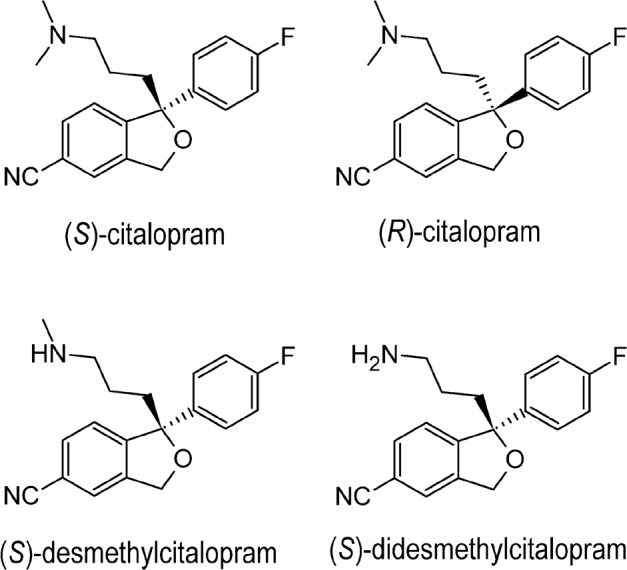
Enantiomers and metabolites of citalopram.

Citalopram is a commonly utilized antidepressive agent of the SSRI family [16, 17]. The structure of citalopram or (*RS*)-1-[3-(dimethylamino)propyl]-1-(4-fluorophenyl)-3H-2-benzofuran-5-carbonitrile is presented in **Figure 1**. Citalopram has 1 asymmetric center and was originally marketed as a racemate (equimolar ratio of (*R*)- and (*S*)-citalopram). As found in numerous studies in vitro and in vivo, (*S*)-citalopram is pharmacologically more active, with (*S*)-desmethylcitalopram [(*S*)-d-citalopram] being active to a smaller extent [18]. Investigations have also indicated (*R*)-citalopram to be therapeutically dormant and to reduce the activity of (*S*)-citalopram [19]. After administering (*RS*)-citalopram, the plasma level of (*S*)-citalopram is roughly a third of the overall drug, although it is uncertain whether the relatively frequent removal of (*S*)-citalopram is attributed to the stereoselective activity of cytochrome P450 (CYP) enzymes in the liver [20,21,22].

An enantioselective study of citalopram and its metabolites in femoral blood from about 50 autopsies using a chiral high-performance LC (HPLC) technique indicated that the *S*:*R* ratio for citalopram was 0.67 ± 0.25 (mean ± standard deviation [SD]) and that for desmethylcitalopram was 0.68 ± 0.20 (mean ± SD). Further, it was shown that the *S*:*R* ratios increase with increasing concentrations of citalopram [23]. “Chiral switch” is an expression used to describe the substitution of an already-accepted-and-sold racemic drug mixture with a pure enantiomer [24, 25]. Citalopram was originally marketed as a racemate, but the difference between the physiological actions of the individual enantiomers resulted in a chiral switch to escitalopram, the pharmacologically active *S*-(+)-enantiomer of racemic citalopram [26, 27]. Presently, citalopram is among the antidepressive agents that are available on the market as both racemates and a single enantiomer.

The metabolism of citalopram by demethylation produces an effective metabolite, desmethylcitalopram, which is around 6 times less effective than (*S*)-citalopram; moreover, the metabolite (*R*)-desmethylcitalopram is about 4-fold more effective than (*R*)-citalopram. Further, the *N*-demethylation of desmethylcitalopram to didesmethylcitalopram (DDCIT) has a high inhibitory efficacy and achieves a greater reduction in plasma levels than the parent drug and its major metabolites (**Figure 1**) [26, 28]. Application of (*S*)-citalopram has many benefits compared with (*RS*)-citalopram, such as enhanced efficacy, lower dosage, and reduction of negative effects of (*R*)-citalopram; this is an ideal model for the advantages of chiral switch [18]. Depending on the amount of (*S*)-citalopram administered (whether as (*S*)-citalopram or as (*RS*)-citalopram), a specified dosage of (*S*)-citalopram was anticipated to be 2 times the dosage of (*RS*)-citalopram [29, 30]. Nevertheless, the outcomes of clinical trials indicated preferable results while using (*S*)-citalopram [31].

An influence of the interaction of (*R*)-citalopram on (*S*)-citalopram, with greater affinity toward the principal binding site and its detachment from the serotonin transporter through an allosteric mechanism, can describe the potential antagonism of (*R*)-citalopram to the effect of (*S*)-citalopram. The behavior of serotonin in coupling to 2 sites (the main and allosteric sites) on the transporter may be accountable for the greater binding and, thus, the higher hindrance of the neurotransmitter by (*S*)-citalopram, because (*S*)-citalopram can be regarded as an allosteric serotonin reuptake inhibitor [32].

In addition, (*R*)-citalopram is a substrate for CYP2D6, and, therefore, hereditary polymorphism and variance in drug quantities are avoided by the administration of (*S*)-citalopram [33]. Findings of randomly assigned, double-blind, placebo-controlled, regulated clinical trials demonstrate that (*S*)-citalopram has a higher efficacy than (*RS*)-citalopram at dose levels that were anticipated to be comparable to (*RS*)-citalopram at doses that resulted in less drug termination [34]. Clinical studies indicate that (*S*)-citalopram, may have a faster onset than (*RS*)-citalopram and that the single enantiomer tends to be safer and more effective than the racemates [35, 36]. However, there has been no definitive proof that individuals with severe depression who adapt well to the racemate benefit by shifting to (*S*)-citalopram.

### Fluoxetine

**Figure 2:**
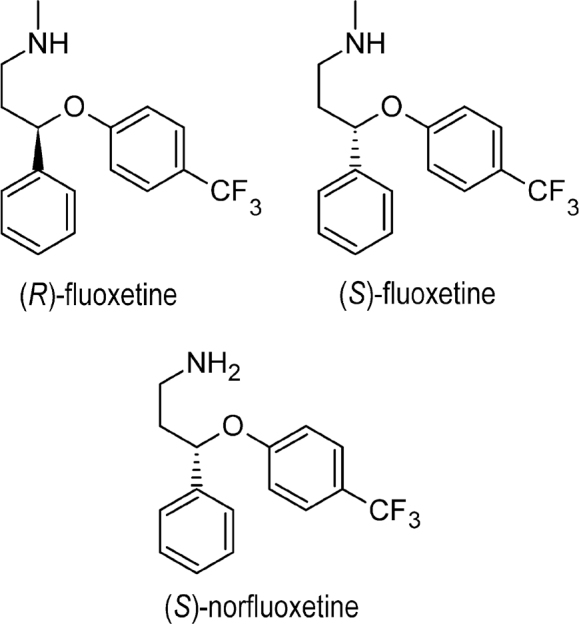
Enantiomers and metabolites of fluoxetine.

Fluoxetine belongs to the important class of antidepressive agents, the SSRIs. Fluoxetine or *N*-methyl-3-phenyl-3-[4-(trifluoromethyl)phenoxy]propan-1-amine was the first SSRI therapy introduced and has been used to treat major depressive disorder, panic disorder, obsessive–compulsive disorder, premenstrual dysphoric disorder, and nervous bulimia [37, 38]. Fluoxetine contains a chiral center (**Figure 2**). Unlike citalopram, fluoxetine could not be developed as a single-enantiomeric form and is still sold as a racemate. Both (*R*)- and (*S*)-fluoxetine display similar activity in blocking serotonin reuptake; however, the 2 enantiomers are metabolized differently [39, 40]. Administration of (*R*)-fluoxetine leads to less-variable plasma concentrations of fluoxetine and its active metabolites than (*RS*)-fluoxetine. The (*R*)-enantiomer and its metabolites inhibit CYP2D6 to a lesser extent than the (*S*)-enantiomer and its metabolites [41]. The racemate of fluoxetine has been observed to be a safe and efficient anti-depressive agent for >15 years, and (*R*)-fluoxetine is not used due to safety issues.

The metabolism of fluoxetine via *N*-demethylation by the CYP enzyme system in the liver is stereoselective, leading toward an active chiral metabolite, norfluoxetine. Norfluoxetine has a pharmacological profile comparable to that of fluoxetine, but achieves different plasma concentrations and leads to the pharmacological effect seen with fluoxetine therapy [42]. Unlike the 2 enantiomers of fluoxetine, the metabolites of (*S*)-fluoxetine [(*S*)-norfluoxetine] and (*R*)-fluoxetine [(*R*)-norfluoxetine] show different efficacies as compared with SSRIs: (*S*)-norfluoxetine is more effective than (*R*)-norfluoxetine. Plasma levels of (*S*)-norfluoxetine in adult patients were higher than levels of (*R*)-norfluoxetine in patients treated with (*RS*)-fluoxetine [42]. (*R*)-Fluoxetine and (*S*)-fluoxetine present distinct metabolic processes, because (*R*)-fluoxetine clearance is around 4-fold higher than that of (*S*)-fluoxetine clearance [43]. The half-lives for the elimination of (*S*)-fluoxetine and (*S*)-norfluoxetine are influenced by the variation in CYP2D6 activity to a higher degree than that of the (*R*)-enantiomers [42, 43]. The stereo-selective metabolism of fluoxetine and norfluoxetine in vitro indicate that *N*-demethylation is also involved in metabolic processes; in addition, CYP2D6, CYP2C9, and—to a smaller extent—CYP2C19 are also involved in *N*-demethylation, with a preference for *R*-norfluoxetine. Conversely, CYP2D6 exhibits greater interaction toward (*S*)-norfluoxetine than (*R*)-norfluoxetine [42, 44]. Therefore, clinical trials have been conducted to evaluate the safety and effectiveness of (*R*)-fluoxetine. However, administration of higher doses of (*R*)-fluoxetine in Phase II clinical trials resulted in a small, but significant, effect on cardiac repolarization, and so the investigations were terminated [37, 45]. (*S*)-Fluoxetine has also been investigated for migraine prophylaxis in clinical trials, but has not obtained U.S. Food and Drug Administration (FDA) regulatory approval to date [37]. Perinatal SSRI exposure produces brain-wide differences in the functional activity of the unborn child during adolescence and adulthood [46].

### Sertraline

In 1991, sertraline was approved by the U.S. FDA for the treatment of severe depression and, in 2003, approved for the management of social anxiety disorder [47]. Sertraline has been reported to be successful in the treatment of generalized anxiety disorder for nearly 20 years [48, 49]. Sertraline or (1*S*,4*S*)-4-(3,4-dichlorophenyl)-*N*-methyl-1,2,3,4-tetrahydronaphthalen-1-amine has been utilized for the diagnosis and treatment of major depression, panic, obsessive–compulsive, and social anxiety disorders [50].

**Figure 3:**
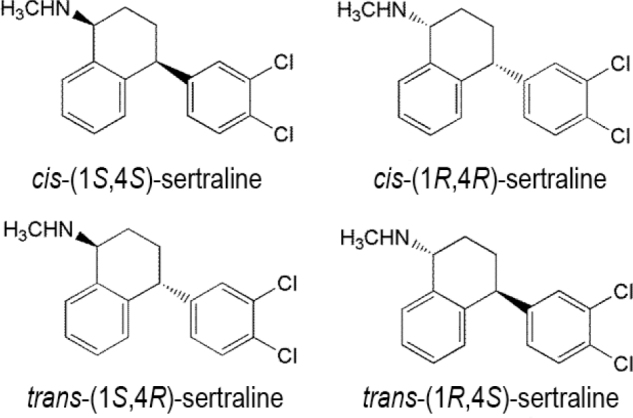
Stereoisomers of sertraline.

The structure of sertraline comprises 2 stereocenters and thus has 4 stereoisomers, but it is sold as a single enantiomer because only the stereoisomer with the 1*S*,4*S* configuration is clinically effective [51]. Across its preparation, substantial amounts of (−)-*cis*-(1*R*,4*R*)-*N*-methyl-4-(3,4-dichlorophenyl)-1,2,3,4-tetrahydro-1-naphthalenemine hydrochloride, *trans*-(1*S*,4*R*), and (1*R*,4*S*)-*N*-methyl-4-(3,4-dichlorophenyl)-1,2,3,4-tetrahydro-1-naphthalenemine hydrochloride are produced, which are considered as impurities (**Figure 3**). The stereoisomer used for the treatment has a configuration (+)-*cis*-(1*S*,4*S*).

Studies conducted to determine the enantiomers of sertraline in Wistar rat plasma 6 h after oral administration of rac-sertraline indicated the presence of *trans* isomers (1*S*,4*R*) and (1*R*,4*S*) in the plasma. This confirmed that only the *cis-*(1*S*,4*S*) and *cis*-(1*R*,4*R*) enantiomers were present in Wistar rats when rac-sertraline was administered. However, in the rat plasma, no sertraline metabolites were found [52]. A pharmacokinetic study of sertraline found the kinetics to be stereoselective in terms of the area under the curve (AUC_0–∞_) (*P* = 0.68), clearance (*P* = 0.68), and maximum serum concentration of a drug (*C*_max_) (*P* = 0.43). Wistar male rats displayed greater plasma levels of *cis*-(1*S*,4*S*)-sertraline. Further, higher AUC_0–∞_ was observed for *cis*-(1*S*,4*S*)-sertraline than for the *cis*-(1*R*,4*R*)-sertraline enantiomer. The disposition of sertraline is enantioselective in male Wistar rats, with a (+):(−) plasma concentration ratio (AUC) near 1.04. The *C*_max_ was also greater for (+)-*cis*-(1*S*,4*S*)-sertraline than for (−)-*cis-*(1*R*,4*R*)-sertraline [52].

### Paroxetine

**Figure 4:**
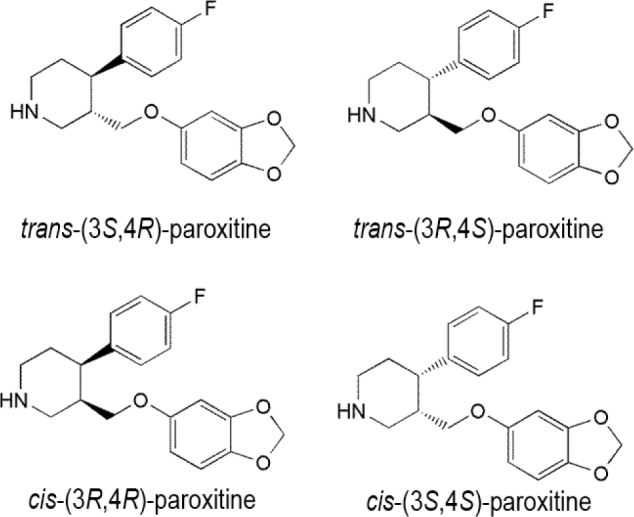
Stereoisomers of paroxetine.

Paroxetine is another SSRI used in the treatment of all kinds of depressive disorders. The paroxetine enantiomer used in therapeutics is (−)-*trans*-(3*S*,4*R*)-3-(1,3-benzodioxol-5-hydroxymethyl)-4-(4-fluorophenyl)piperidine (**Figure 4**); it is composed of 2 asymmetric centers and can form 4 stereoisomers, of which 2 have *trans* and 2 have *cis* conformations. Paroxetine is sold as an individual enantiomer, that is, the (−)-*trans*-(3*S*,4*R*) stereoisomer, which is therapeutically more active than the other stereoisomers [53]. However, in drug preparations, its inactive enantiomer, (+)-*trans*-paroxetine, may also be present [54].

## Serotonin–norepinephrine reuptake inhibitors

SNRIs are a class of antidepressive agents used for the diagnosis and treatment of depression and other cognitive disorders, such as anxiety, obsessive–compulsive disorder, attention deficit hyperactivity disorder (ADHD), and chronic pelvic pain. SNRIs affect 2 neurotransmitters, namely, serotonin and norepinephrine, in the brain. Due to their compound effect, both their efficiency and tolerability tend to be better than those of the SSRIs.

### Venlafaxine

**Figure 5:**
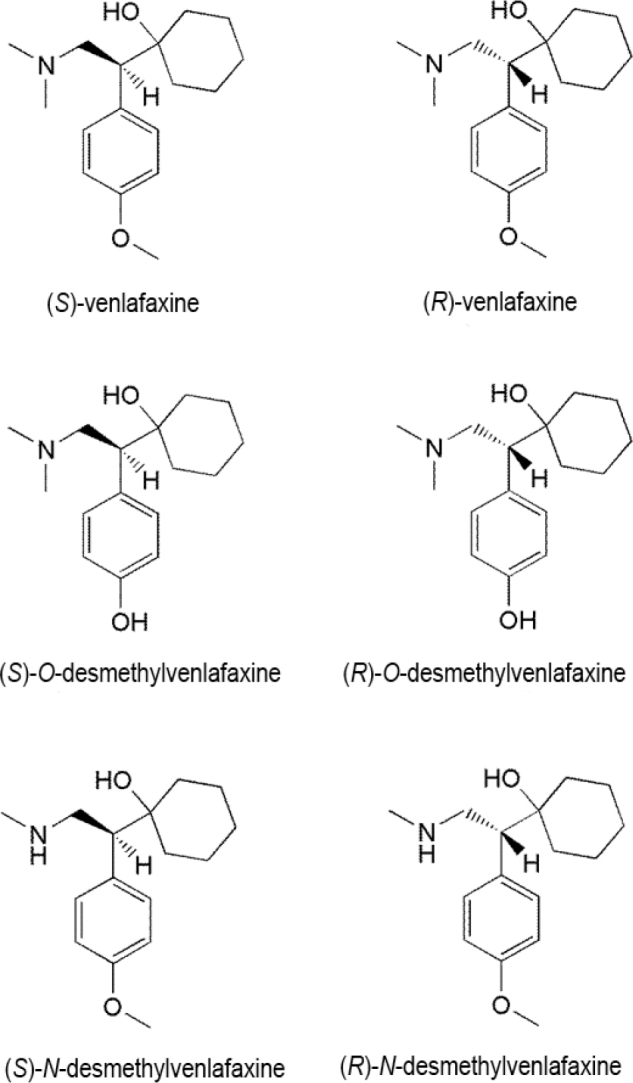
Enantiomers and metabolites of venlafaxine.

Venlafaxine was first manufactured in the early 1980s and is known to suppress the reuptake of serotonin and noradrenaline with low efficacy [54]. Venlafaxine has been demonstrated to function in animal models of anxiety in vivo and has less interaction with postsynaptic muscarinic or histaminergic receptors [54]. Venlafaxine is expected to have a higher tolerability profile than tricyclic antidepressive agents and is among the most widely used drugs in the SNRI category [44,45,46,47,48,49,50,51,52,53]. Venlafaxine is used as an antidepressive agent and is administered as a racemic mixture consisting of the enantiomers (*S*)- and (*R*)-venlafaxine in equal quantities [55]. Enantiomers of venlafaxine or 1-[2-(dimethylamino)-1-(4-methoxyphenyl)ethyl]cyclohexan-1-ol (**Figure 5**) display different therapeutic behaviors, and they affect neurotransmitters differently. The (*R*)-form of venlafaxine is a potent inhibitor of the reuptake of serotonin noradrenaline, whereas the (*S*)-form is more selective in suppressing the reuptake of serotonin only [56, 57]. However, both enantiomeric forms exhibit practical therapeutic activity in treating depression, although they interact with the 2 neurotransmitters in different ways [58].

(*S*)-Venlafaxine displays greater potency toward CYP2D6 as an inhibitor than (*R*)-venlafaxine in vitro; it is selectively metabolized at clinical doses by CYP2D6 in humans, whereas the opposite occurs at high doses [59, 60]. Venlafaxine is primarily metabolized in the liver to *O*-desmethylvenlafaxine, *N*-desmethylvenlafaxine, *N*,*O*-didesmethylvenlafaxine, and potent metabolites, which are also chiral compounds. The major metabolic route in humans is the *O*-demethylation of racemic venlafaxine to *O*-desmethylvenlafaxine, while *N*-demethylation of the alkylamino side chain in *N*-desmethylvenlafaxine and the removal of both *O*-methyl and *N*-methyl groups in *N*,*O*-didesmethylvenlafaxine are the minor routes. Among these, the *O*-desmethyl derivative maintains similar pharmacological interaction as the parent drug, whereas the *N*-desmethyl derivative is also effective, although it is a weaker inhibitor of serotonin and norepinephrine reuptake than venlafaxine [61]. The (*R*)- and (*S*)-enantiomeric forms of the metabolites preserve their parent drug's respective effectiveness to inhibit serotonin and norepinephrine reuptake [61].

### Duloxetine

**Figure 6:**
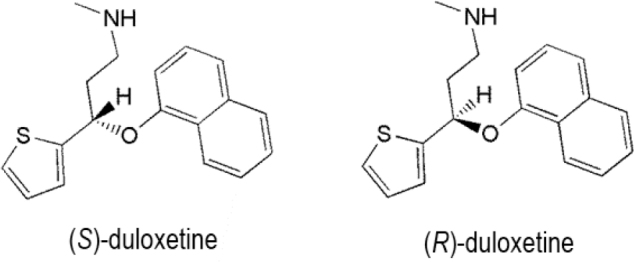
Enantiomers of duloxetine.

Duloxetine or (+)-*S*-*N*-methyl-3-naphthalene-1-yloxy-3-thiophen-2-ylpropan-1-amine (**Figure 6**) is an important SNRI used to treat major depression, general anxiety, fibromyalgia, and chronic pain [62, 63]. It may also be used to treat certain medical problems such as urinary incontinence, in addition to the treatment of psychiatric disorders [64]. It has a chiral center and is applied as a single enantiomer in medication, namely, as (*S*)-duloxetine. Both the enantiomeric forms of duloxetine effectively inhibit norepinephrine and serotonin reuptake, although the (*S*)-form is 2-fold more active than the (*R*)-form and is sold as a single-enantiomeric form for clinical therapy [65, 66]. Studies on the binding of duloxetine enantiomers to human serum albumin indicate that the binding constant for the (*R*)-enantiomer is greater than that for rac-duloxetine. Moreover, the effect of pH on the binding constants showed that at pH 7.4, both (*R*)-enantiomer and rac-duloxetine showed similar binding, but at higher pH (8.5), (*R*)-duloxetine showed better binding ability than racduloxetine [67].

The direct enantioseparation of duloxetine and its *R*-enantiomer has been achieved by HPLC using hydroxypropyl-β-cyclodextrin as a chiral selector and vancomycin as a chiral stationary phase (Chirobiotic V), with a limit of detection of 0.06 μg per mL [68].

### Milnacipran

**Figure 7:**
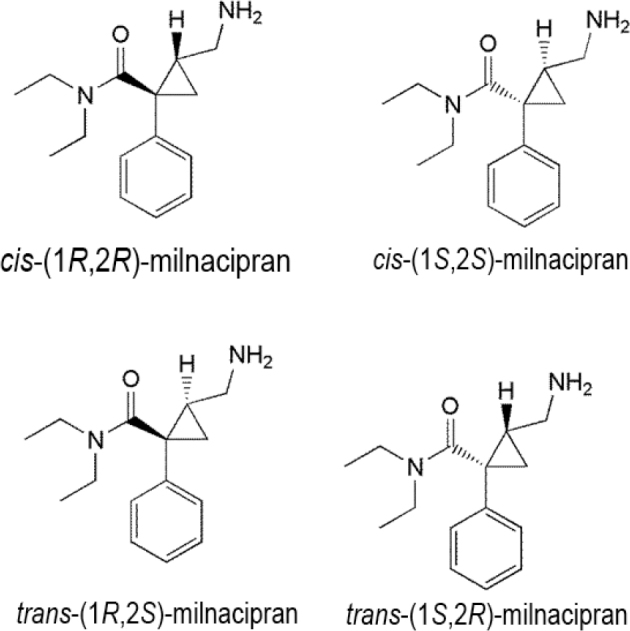
Stereoisomers of milnacipran.

Milnacipran is an SNRI accepted for the treatment of fibroma by the U.S. FDA and for the treatment of depression by agencies elsewhere [69]. Milnacipran or (2-(aminomethyl)-*N*,*N-*diethyl-1-phenylcyclopropane-1-carboxamide) possesses 2 chiral centers in its structure (**Figure 7**) and is available as an equimolar mixture of the *cis* isomers with the following conformation: d-milnacipran (1*S*,2*R*) and l-milnacipran (1*R*,2*S*). Pharmacokinetic studies in humans [70, 71] have demonstrated a median peak plasma time (*t*_max_) of 2 h. Milnacipran is steadily absorbed from the gastrointestinal system. The absolute oral bioavailability of milnacipran is large (85%), and diet does not affect absorption. The plasma half-life is around 8 h, and approximately 50%–60% of milnacipran is excreted unaltered in the urine. When administered orally, nearly the entire dose (about 93%) is eliminated steadily in the urine.

Elimination of the milnacipran carbamoyl *O*-glucuronide metabolite in urine (about 19% of the dose) is primarily as a metabolite of l-milnacipran carbamoyl *O*-glucuronide (about 17% of the dose); approximately 8% of the dose excreted in urine is a metabolite of *N*-desethylmilnacipran. No significant quantities of extra metabolites are secreted in the urine. Comparable plasma levels of milnacipran and l-milnacipran carbamoyl *O*-glucuronide metabolite have been detected after medication, and the highest possible plasma level of l-milnacipran carbamoyl *O*-glucuronide metabolite at 4 h after dosing was 234 ng per mL. Low levels of *N*-desmethylmilnacipran and d-milnacipran carbamoyl *O*-glucuronide metabolites (<25 ng per mL of milnacipran) have been reported in the plasma [70].

## Norepinephrine reuptake inhibitors

NRIs inhibit norepinephrine (noradrenaline) and epinephrine reabsorption by neurons in the brain. Inhibiting the reabsorption influences the neuronal activity that certain neurotransmitters provide, enhancing cognition and concentration. NRIs have many applications, such as for the treatment of sleep disturbances, major depression, and ADHD. The adverse effects of NRIs include headache, digestive discomfort, and increased heart rate. Overdose of these drugs can lead to severe physiological complications, including anxiety and brain trauma [71].

### Reboxetine

**Figure 8:**
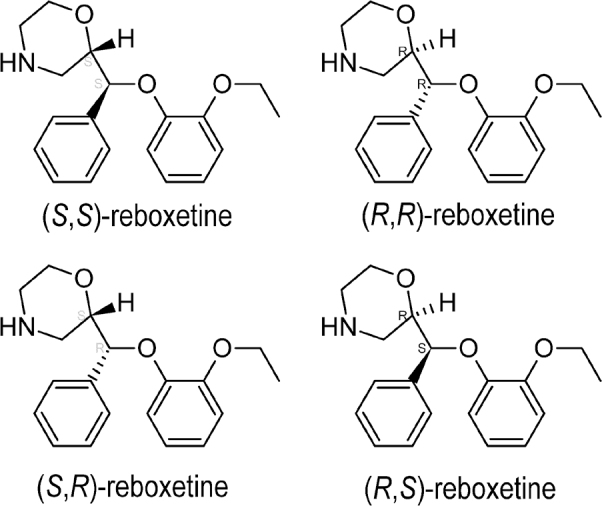
Stereoisomers of reboxetine.

Reboxetine or (2*RS*)-2-[(*RS*)-(2-ethoxyphenoxy)-phenylmethyl]morpholine is the most effective NRI medication used to manage anxiety. Reboxetine has 2 chiral centers (**Figure 8**) and can therefore appear as a racemate; nevertheless, during enantioselective synthesis, mostly (*R,R*)-, and (*S,S*)-reboxetine are formed [72]. Therefore, it is available as a racemate in clinical preparations. However, the 2 enantiomers show substantially different pharmacological properties [73, 74]. For instance, the *C*_max_ and AUC_0–*t*_*z*__ of (*R,R*)-reboxetine are greater than those of (*S,S*)-reboxetine [75]. Conversely, several reports indicate that (*S,S*)-reboxetine is a more active NRI than (*R,R*)-reboxetine [76] and is accountable for the vasomotor and adverse effects of reboxetine on the heart [77].

Receptor-binding models in vitro and in vivo indicate that (*S,S*)-reboxetine is the most active NRI [78], even though its plasma levels are almost half after either injectable or oral administration [79]. The activity is not a consequence of stereoselective metabolism, because CYP3A4 acts on both the enantiomers to about the same extent in vitro [80, 81]. The enantiomers exhibit identical half-lives, irrespective of the route of administration [74, 81]. The difference in plasma levels between the enantiomeric forms is likely due to better renal clearance of (*S,S*)-reboxetine [74, 82]. The predicted distribution of reboxetine enantiomers following an injectable dose is approximately 2.4-times higher. The difference indicates that enantioselective disposition of reboxetine can result from an association or redistribution with enantioselective tissue. Reboxetine is available as a racemic drug with an explicitly active enantiomer, whereby its enantioselective nature has impaired the accurate description of the dosage–effect interaction [77].

## Norepinephrine–dopamine reuptake inhibitors

### Bupropion

**Figure 9:**
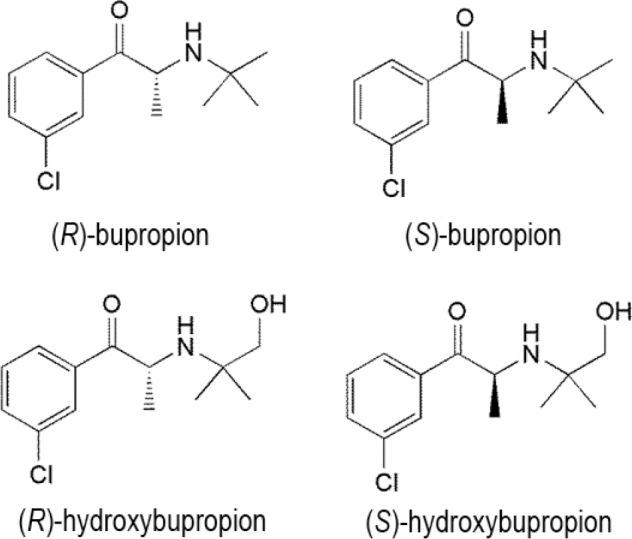
Enantiomers and metabolites of bupropion.

Bupropion ((±)-2-(*tert*-butyl amino)-1-(3-chlorophenyl)propane-1-one) is an important member of the NDRI class and has 1 chiral center (**Figure 9**). It is used clinically as a racemic mixture [81, 82] and as its pure enantiomers [83]. The (*R*)-enantiomer is more effective than the (*S*)-enantiomer [84]. Bioactivation of bupropion is catalyzed predominantly by stereoselective CYP2B6 and CYP2B6-catalyzed bupropion hydroxylation [85].

Investigations in vitro have established that bupropion may be a noncompetitive, functional inhibitor of nicotinic acetylcholine receptors [86]. The stereoselective pharmacokinetics of bupropion and its metabolites, namely, (*R*)-bupropion:(*S*)-bupropion and (1*R*,2*R*)-:(1*S*,2*S*)-threohydrobupropion, and their ratios are probably indicative of greater presystemic metabolism of (*S*)- versus (*R*)-bupropion by carbonyl reductases. Remarkably, the apparent renal clearance of (2*S*,3*S*)-hydroxybupropion is nearly 10 times greater than that of (2*R*,3*R*)-hydroxybupropion [87]. Bupropion is metabolized into 3 active metabolites, namely, hydroxybupropion, erythrohydrobupropion, and threohydrobupropion (**Figure 9**), which further metabolize into ineffective metabolites and are eventually eliminated via the urinary tract [88]. Hydroxybupropion is the main metabolite of bupropion detected in human plasma [89] and is obtained by the hydroxylation of its *tert*-butyl group and amino alcohol isomers and the consequent morpholino ring formation [90]. The *C*_max_ and AUC_0–1_ for (*S*)-bupropion were found to be 3 and about 2 times higher respectively, than those of (*R*)-bupropion [90]; whereas, the *C*_max_ and AUC_0–∞_ for (*R*,*R*)-hydroxybupropion were 11 and 7 times higher respectively, than for (*S*,*S*)-hydroxybupropion. Further, the AUC ratio of metabolite to parent plasma was found to be 12–15 times higher [84]. By contrast, a study of bupropion in patients with kidney disease found that its pharmacokinetics is stereoselective. In the context of kidney disease, the time for rac-bupropion, (*R*)-bupropion, and (*S*)-bupropion to display *t*_max_ was 4 h [90]. While the *t*_max_ for hydroxybupropion and (*R*,*R*)-hydroxybupropion was found to be around 16 h, (*S*,*S*)-bupropion showed a *t*_max_ of 8.8 h and demonstrated a faster absorption [89]. The AUC of (*R*)- and (*S*)-bupropion were 30% and 70% of the AUC of racbupropion, respectively; thus, indicating that (*S*)-bupropion is the main enantiomer distributed in normal patients [84, 87]. The AUC of (*R*,*R*)-hydroxybupropion and (*S*,*S*)-hydroxybupropion were 95% and 5% of the AUC of hydroxybupropion, respectively; thus presenting (*R*,*R*)-hydroxybupropion as the prime metabolite [90].

## Monoamine oxidase inhibitors

**Figure 10:**
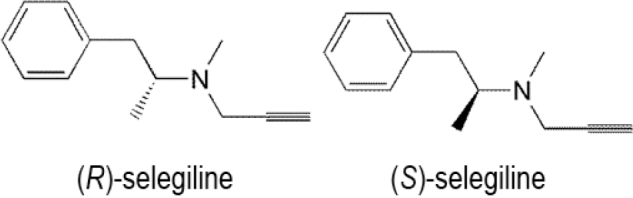
Enantiomers of selegiline.

Monoamine oxidase inhibitors are a family of drugs that suppress one or both monoamine oxidase enzymes namely: monoamine oxidase A and monoamine oxidase B. One of the important examples of this class is selegiline ((2*R*)-*N-*methyl-1-phenyl-*N*-prop-2-ynylpropan-2-amine) (**Figure 10**). Selegiline is metabolized in the kidney by 3 different pathways, namely, *N*-dealkylation, β-carbon hydroxylation, and ring-hydroxylation, producing 9 metabolites, (*R*)-desmethylselegiline, (*R*)-methamphetamine (MA), (*R*)-amphetamine, (*1S,2R*)-norephedrine, (*1R,2R*)-norpseudoephedrine, (*1S,2R*)-ephedrine, (*1R,2R*)-pseudoephedrine, (*R*)-*p*-hydroxyamphetamine, and (*R*)-*p*-hydroxymethamphetamine, which are encountered in urine along with unmetabolized drug. The major metabolite is (*R*)-methamphetamine. However, during metabolism, no racemization takes place and β-carbon hydroxylation demonstrates stereoselectivity [91].

After oral administration of selegiline tablets, (*R*)-desmethylselegiline, methamphetamine, and amphetamine were found as the (−)-isomers in the urine and plasma. Nearly 40% of orally ingested selegiline was excreted in urine within the first 2 d after dosing as 3 metabolites, namely, (*R*)-desmethylselegiline, methamphetamine, and amphetamine. Additionally, no parent selegiline was found in urine or plasma samples [91]. Conversely, (*R*)-methamphetamine and amphetamine were only found as the (+)-enantiomers in the urine of (*R*)-methamphetamine abusers. For abusers of selegiline, (−)-(*R*)-desmethylselegiline, a selegiline metabolite, is not an appropriate predictor because (−)-(*R*)-desmethylselegiline is quickly removed from the urine, and only 1% of the dose is secreted. The mean residence times of (−)-(*R*)-desmethylselegiline in plasma and urine are 5–20 times lower than those of (−)-methamphetamine or (−)-amphetamine [92].

## Tetracyclic antidepressants

**Figure 11:**
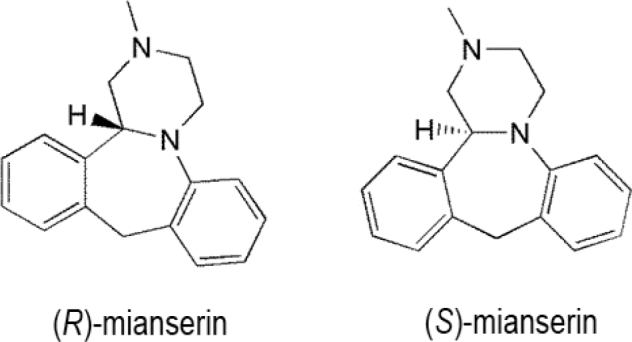
Enantiomers of mianserin.

An important example of this tetracyclic antidepressant class is mianserin (5-methyl-2,5-diazatetracyclo[13.4.0.02,7.08,13] nonadeca-1(19),8,10,12,15,17-hexaene), which contains 1 chiral center (**Figure 11**) and functions by blocking presynaptic α2-adrenergic autoreceptors. Mianserin is produced as a racemate, and (*S*)-mianserin is more potent than (*R*)-mianserin as an inhibitor of the reuptake of noradrenaline [93]. In several animal behavioral experiments, it corresponds with antidepressive action. The mechanism of action of mianserin is stereoselective and essentially favors (*S*)-mianserin [94, 95]. Nevertheless, both enantiomers have identical activities for the antagonism toward α2-adrenergic heteroreceptors on 5-hydroxytryptaminergic (5-HT) nerve terminals [94]. Mianserin enantiomers still vary remarkably from one another in their capability to transport the bound radioligands to distinct 5-HT receptor subtypes [96, 97]. In a variation, pharmacological evaluations found that the sedative (seemingly expected from the antihistaminic potency of mianserin) effects are identical for the single enantiomers of the drug. Some investigations have indicated noteworthy metabolic distinctions between the 2 enantiomers of mianserin. Mianserin is widely metabolized and its excretion in human urine is unaffected (only about 5%). The major paths for the metabolism of mianserin in humans are aromatic hydroxylation, *N*-oxidation, and *N*-demethylation [98]. (*S*)-Mianserin is considered an effective antidepressant; however, its 2 enantiomers display identical sedative effects [99]. Desmethylmianserin and 8-hydroxymianserin maintain antidepressant activities, but are less sedative than mianserin, whereas mianserin-*N-*oxide tends to be a comparatively inactive metabolite [100]. Thus, the 2 enantiomers display substantially different enantioselective variations in metabolism [101, 102]. Studies conducted with mianserin or the active metabolite, desmethylmianserin, have found coadministration of benzodiazepines is not an important factor in deciding the dosage of mianserin [101]. In an analysis using human liver microsomes and 8 recombinant CYP isozymes, CYP2D6 has been shown to mediate the 8-hydroxylation of the 2 enantiomers of mianserin, although CYP1A2 catalyzes the *N*-demethylation of both enantiomers in equal amounts, whereas *N*-oxidation favored for *S*-mianserin [103]. CYP3A is implicated in each of the stereoselective mianserin processes to some degree. The mean values for the ratio of the maximum rate of the CYP-catalyzed reaction to the substrate concentration that yields the half-maximal velocity (Michaelis constant) (*V*_max_:*K*_m_) of 8-hydroxylation and *N*-oxidation were higher for (*S*)-mianserin than for (*R*)-mianserin, while the reverse was observed for *N*-demethylation; this reverse enantioselectivity for *N*-demethylation and *N*-oxidation mirrored previous results in rats and mice and human hepatic microsomes [100, 101, 104]. Significant interindividual differences in the percentages of the 2 enantiomers of mianserin and *N*-desmethylmianserin have been already identified in various plasma samples [105]. In an analysis of weak and comprehensive debrisoquin and mephenytoin metabolizers, the disappearance of both mianserin and *N*-desmethylmianserin depended on CYP2D6 activity, and CYP2D6-dependent removal of mianserin demonstrates significant enantioselectivity for both (*S*)-mianserin and *N*-desmethylmianserin [106].

## Noradrenergic and specific serotonergic antidepressants

NASSAs, such as mirtazapine, have a double mode of action, which increase the 5-HT and noradrenaline concentrations in the sarcoplasmic reticulum to within a normal range. NASSAs act by blockade of both α_2_-auto- and α_2_-heteroreceptors controlling noradrenaline and 5-HT release.

**Figure 12:**
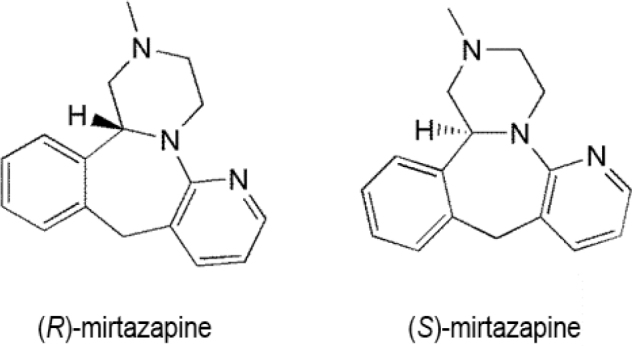
Enantiomers of mirtazapine.

Mirtazapine (**Figure 12**) is a recently developed noradrenergic and powerful serotonergic antidepressive agent commonly administered as a racemate despite the multiple therapeutic actions of the enantiomers. Both stereoisomers have pharmacological effects that seem to predict antidepressant activity. Its (*S*)-form is the more powerful antagonist of receptors of the type α_2_-autoreceptor and 5-HT type-2, while the (*R*)-enantiomer primarily acts on α_2_-heteroreceptors and 5-HT type-3 receptors [107]. Mirtazapine enantiomers are metabolized predominantly by hydroxylation, demethylation, and *N-*oxidation reactions of various CYP enzymes [108].

## Conclusions

Most antidepressive agents contain one or more asymmetric centers in their structure. The enantiomers of antidepressive agents differ substantially in their pharmacodynamic and pharmacokinetic properties. Enantiomers show different affinities toward protein binding, clearance, and stereoselective metabolism. Compositions containing less-active enantiomers hinder pharmacological studies regarding drug disposition and diagnostic effects related to dosage levels. The use of pure enantiomers provides potential advantages, including: (i) decreasing the overall quantity of the allocated drug required to produce a therapeutic effect; (ii) an increased therapeutic effect via enhanced receptor selectivity and efficiency; (iii) diminished detrimental effects; (iv) reduced interindividual inconsistency, and (v) diminished drug–drug binding associations and interactions. Because of the advantages of using pure enantiomers, efforts are made to introduce pure enantiomers of drugs. There are various antidepressants available as pure enantiomers, including (*S*)-citalopram, (*R*)-fluoxetine, (3*R*,4*R*)-paroxetine, and (*S*)-duloxetine. Nonstereoselective pharmacokinetic studies are frequently insufficient to explain the fate of chiral drugs prescribed as racemates. Stereoselectivity is inherently essential in the development of a chiral drug at every phase, and its neglect can inevitably lead to misinterpretation of pharmacokinetic and pharmacodynamic profiles. However, it has become evident that the definition of a chiral medication of effective and inactive stereoisomers is an overgeneralization. The distomer could have a sequence of impacts ranging from eutomer antagonism to explicit tolerance or may even have a substantial impact, which can provide a benefit over the use of racemates. Each medication must be treated individually in its usage as a standard stereoisomer formulation. Many unexamined forms of stereoselectivity could be of clinical importance throughout the metabolism of the drug.

In the production of psychoactive substances, chirality seems to be an essential component because enantiomers frequently display significant variations in their pharmacological effects. This review describes the therapeutic psychopharmacological consequences of stereoisomers of antidepressants as a target. In several instances; a deeper interpretation of stereochemistry would enhance the clinical results. For example, the racemic combination of citalopram is beneficial for depression, anxiety, and obsessive–compulsive tendencies. Nonetheless, escitalopram, or (*S*)-citalopram, is at least 2 times as active as racemic citalopram as a serotonin reuptake inhibitor, meaning that it could be administered at a lower dosage, thus providing an enhanced therapeutic index and a better safety profile with decreased drug interactions. Data from clinical trials support those benefits. Prolonged assessment of the stereochemistry of antidepressant drugs will improve the interpretation of dose–response relationships and may explain the effects of the stereoselective pharmacological properties of drugs in various disorders, gene mutations, pregnancy, sex, and age. Thorough interpretation of the future of chiral antidepressants and the factors influencing their enantioselective behavior and actions should offer a reasonable basis for their extended usage in a wide range of patients.

The growing development of enantiomerically pure medications aims to provide physicians with better, safer, more widely tolerated, and more effective medicines for patients. Practitioners are responsible for familiarizing themselves with the essential principles of the chiral pharmaceutical products mentioned in this article, particularly because each enantiomer of a specific chiral substance can have a specific pharmacological profile, and a pure enantiomer drug composition can have unique characteristics compared with those of the given drug's racemic formulations. When both enantiomers and a racemate dosage are accessible, clinical evidence and practical skills can be used to assess the suitability of any drug formulation. Advances in scientific methods to distinguish racemate drugs from their pure enantiomers will facilitate the process.

Efforts to establish formulations for the pure eutomer are motivated by the stereoselective pharmacokinetics and pharmacodynamic scale of the same. Such formulations may be more effective, have less or less-severe negative impacts, and have fewer drug interactions than the racemic mixture. Previously, single enantiomers were costly to produce; however, with recent developments in stereospecific synthesis and evaluation, single enantiomer compositions have become more reasonably priced, and enantiomerically pure medications are becoming much more prevalent. Recent advances in the field of antidepressive drug production have resulted in many new medications that include potentially major deviations from their racemic equivalents.
